# Characterization of the Gut Microbiome and Resistomes of Wild and Zoo-Captive Macaques

**DOI:** 10.3389/fvets.2021.778556

**Published:** 2022-01-24

**Authors:** Ting Jia, Wei-Shan Chang, Vanessa R. Marcelino, Sufen Zhao, Xuefeng Liu, Yuyan You, Edward C. Holmes, Mang Shi, Chenglin Zhang

**Affiliations:** ^1^Beijing Key Laboratory of Captive Wildlife Technologies, Beijing Zoo, Beijing, China; ^2^Sydney Institute for Infectious Diseases, School of Life and Environmental Sciences and School of Medical Sciences, The University of Sydney, Sydney, NSW, Australia; ^3^Department of Molecular and Translational Sciences, Monash University, Clayton, VIC, Australia; ^4^Centre for Innate Immunity and Infectious Diseases, Hudson Institute of Medical Research, Clayton, VIC, Australia; ^5^School of Medicine, Sun Yat-sen University, Guangzhou, China

**Keywords:** monkey, microbiome, antimicrobial resistance gene, adenoviruses, captive primates, metagenomic

## Abstract

Rhesus macaques (*Macaca mulatta*) are the most widely distributed species of Old World monkey and are frequently used as animal models to study human health and disease. Their gastrointestinal microbial community likely plays a major role in their physiology, ecology and evolution. Herein, we compared the fecal microbiome and antibiotic resistance genes in 15 free-ranging and 81 zoo-captive rhesus macaques sampled from two zoos in China, using both 16S amplicon sequencing and whole genome shotgun DNA sequencing approaches. Our data revealed similar levels of microbial diversity/richness among the three groups, although the composition of each group differed significantly and were particularly marked between the two zoo-captive and one wild groups. Zoo-captive animals also demonstrated a greater abundance and diversity of antibiotic genes. Through whole genome shotgun sequencing we also identified a mammalian (simian) associated adenovirus. Overall, this study provides a comprehensive analysis of resistomes and microbiomes in zoo-captive and free-ranging monkeys, revealing that semi-captive wildlife might harbor a higher diversity of antimicrobial resistant genes.

## Introduction

Rhesus macaques (*Macaca mulatta*) are a species of Old World Monkey with a wide geographic distribution. Because of their close phylogenetic relationship with humans, they are extensively used as biomedical models for understanding human disease. A handful of publications have demonstrated that the non-human primate (NHP) gut microbiome is shaped by diet, evolutionary features, age, sex, geographical habits (1–4), and notably captivity, indicating that human-mediated life styles and living locations could alter the gut-associated microbial communities of primates (5). Many previous studies have investigated the impact of captivity, diet and anthropogenic activity on microbiome composition. For example, Clayton et al. examined the gut microbiome in different species of NHPs such as douc and howler monkeys, showing that diversity of native gut microbial taxa was reduced among the captive groups (5). In black howler monkeys, the environmental and dietary changes associated with captivity had a major impact on intestinal microbial methanogenesis (6). In contrast, similar bacterial compositions were observed in wild vs. captive chimpanzees (7).

Driven by advances in next-generation sequencing technologies, microbiome and resistome focused studies of are increasing importance, expanding our knowledge of microbial communities and their interactions with humans, animals and the environment. Amplicon sequencing is sufficient for family-level and genus-level bacterial classification, although the variation captured by 16S sequencing is insufficient for strain-specific identification (8). Additionally, metagenomic approaches provide a means to characterize non-bacterial microbes, including viruses and eukaryotic pathogens (9). Recent studies have also revealed that host-associated intestinal microbiota may impact viral susceptibility and the ensuing host immune responses (10, 11). The widespread use of antibiotic agents in veterinary and human medicine has revolutionized the therapeutic options of bacterial infection, although at the same time it has increased the selection pressure for the rapid emergence and evolution of antimicrobial resistance (12).

Herein, we used both 16S rRNA and whole genome shotgun DNA sequencing approaches to identify the differences of fecal microbial composition and resistome between zoo-captive and wild rhesus monkeys in China. Our results provide important insights on the impact of captivity on microbial diversity and antimicrobial resistance properties.

## Materials and Methods

### Animal Ethics Statements

This study was approved by the Beijing Municipal Committee of Animal Management before sample collection. All experiments were performed in accordance with the approved guidelines and regulations under approval number #SYSU-IACUC-MED-2021-B0123.

### Study Sites and Sampling Information

This study was conducted from July to August in 2014 at Shennongjia Forestry District natural reserves (SR), a zoo located in Beijing (BR) and a wildlife zoo located in inner Monglia (ER). All fecal specimens of rhesus monkeys (*Macaca mulatta*) were collected following defection at three sampling locations: one wild (SR), one semi-captive (ER), and a zoo-captive population (BR). Details of the sample collection sites, sample groups and food usage are presented in Table 1. DNA extraction of the fecal samples was performed using the TruSeq™ DNA Sample Prep Kit (Illumina) following the manufacturer's instructions.

**Table 1:** Sample location and size of zoo-captive and wild rhesus monkeys.

**Group name**	**Type**	**Location**	**Sample size**	**Latitude**	**Food source**
BR	Zoo-captive	Beijing	24	39.94°N	Potatoes, fruits, vegetables, steamed corn bread
ER	Semi-zoo-captive	Inner Mongolia	57	39.8°N	Fruits, vegetables, steamed corn bread
SR	Free-ranging	Shennongjia Forestry District natural reserves	15	31.46°N	Wild plants

### Comparisons of Bacterial Composition and Diversity Using 16S RRNA Sequencing

Fecal samples from each monkey were subject to 16S rRNA amplicon sequencing. The V3–V4 hypervariable regions of the bacterial 16S ribosomal RNA (rRNA) gene were amplified using barcoded primers, 341F- 5′-CCTACACGACGCTCTTCCGATCTN (barcode) CCTACGGGNGGCWGCAG-3′ and 805R-5′-GACTGGAGTTCCTTGGCACCCGAGAATTCCA (barcode) GACTACHVGGGTATCTAATCC-3′, according to the Illumina 16S Metagenomic Sequencing Library Guide. The amplicons generated were sequenced on an Illumina HiSeq platform in a 2 × 250 paired-end mode. All sequencing and library preparation procedures were performed by Sangon Biotech (Beijing, China).

The raw amplicons generated were screened, trimmed, filtered, denoised, and chimera-depleted using QIIME2 version 2018.2 (http://qiime.sourceforge.net). Short, ambiguous sequences and chimeras deriving from the PCR process were removed using DADA2 plugins. Sequences were clustered into Operational Taxonomic Units (OTUs) and then assigned to bacterial sequences with at least 99% similarity to representative sequences from the SILVA 132 database (http://www.arb-silva.de/). For statistical analysis, all the sequences were rarefied to 1,112 reads for the downstream analysis. For each sample, the relative abundance of each bacterium identified was expressed as the percentage of total reads. QIIME2 was applied to profile the taxonomy of microbial composition in each group and to calculate alpha diversity matrices (including ACE, Shannon diversity index and Simpson index) (13, 14). To evaluate the variation between different groups, beta-diversity distance matrices (including Bray-Curtis distances, weighted and unweighted UniFrac values) were performed using rarefied data sets, and subsequently principal coordinate analysis (PCoA) was conducted to visualize the dissimilarities in the fecal bacterial communities among different groups of rhesus monkeys.

### Fecal Microbiome Characterization

All reads from the high-throughput DNA sequencing data were mapped to reference genomes of *Macaca mulatta* (NCBI txid:9544) using Bowtie2 (15) to remove genetic material of host origin after quality-trimming by Trimmomatic (16). To profile the bacterial results from microbial composition, CCMetagen (17) was used for taxonomic annotation against nt database. To screen for viruses, host-filtered reads from the metagenomic sequencing data sets were assembled using MEGAHIT (18) then compared against the entire nr database in GenBank using Diamond BlastX e value <10^5^) (19). Any viral reads and contigs identified by Blast were then extracted and reassembled using the assembler implemented in Geneious v.11. This process identified abundant adenovirus sequences that were then reassembled into an entire adenovirus genome. This genome was then translated into amino acid sequences for gene annotation and functional prediction using Conserved domain databases (CDD).

The assembled sequences were then aligned using the MAFFT version 7 with implemented E-INS-I algorithm (20). Conserved domains within the E1A and 100k protein of adenoviruses were used for subsequent phylogenetic analyses. After removing all ambiguously aligned regions using TrimAl (21), the final lengths of E1A and 100K protein alignments were 832 and 1,379 amino acid residues, respectively. Phylogenetic trees of these data were inferred using the maximum likelihood method (ML) implemented in PhyML version 3.0, employing a Subtree Pruning and Regrafting topology searching algorithm. Statistical support for specific groupings in the tree was assessed using the approximate likelihood-ratio test (aLRT) with a Shimodaira-Hasegawa like procedure with 1,000 replicate bootstrap. The phylogenetic trees were visualized using the FigTree program (http://tree.bio.ed.ac.uk/software/figtree).

### Detection of Antimicrobial Resistance Genes

To determine the presence of putative antimicrobial resistance (AMR) genes in the data, we analyzed the shotgun sequencing data using the KMA program (22) combined with the ResFinder reference database (23). To reduce false-positive results, genes were only considered in downstream analyses when *p*-values for the conclave score were lower than 0.05, only two genes were excluded due to their *p* > 0.05 (22). We also excluded the blaTEM116 gene which has been previously identified as a common laboratory contaminant (24). AMR diversity and abundance was visualized in R with the package *ggplot2*.

## Results

### Overall Characterization of 16S and Shotgun DNA Sequencing Results

The 16S rRNA amplicon sequencing generated a total of 2,572,794 reads and 2,680 OTUs. The total number of raw reads across all groups from the high-throughput shotgun DNA sequencing data was 1,425,675,194. Rarefaction curves showed a similar trend in all three populations. Observed numbers of OTUs (Observed_OTUs), an indicator of alpha diversity, is a qualitative measure of community richness. By this metric, the population of SR (wild monkeys) harbored the highest numbers of OTUs among all three groups under the same sequencing depth (orange: SR; blue: BR; cyan: ER) (Supplementary Figure 1A). The Shannon-Wiener curves showed that the samples from all groups had plateaued (Supplementary Figure 1B). The rarefaction curves indicate that sequencing depth was sufficient to capture the bacterial diversity in all samples (Supplementary Figure 1).

### Association Between Bacterial Richness and Diversity and Animal Captivity

**Figure 1:**
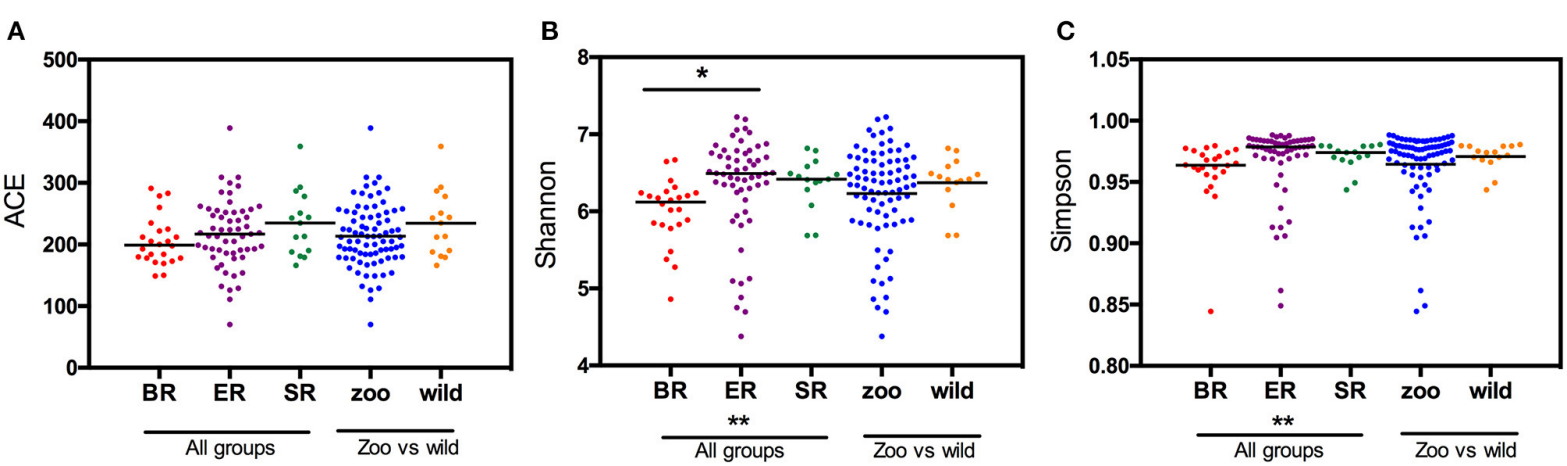
Estimated OTUs richness and diversity index in different groups of monkeys. The index of OTUs richness in different groups was estimated using ACE **(A)** metrics. To estimate OTU diversity, Simpson's index **(B)** and Shannon's index **(C)** were performed. No significant statistical differences in ACE (*p* = 0.23) between the three groups was obtained using Kruskal-Wallis tests. Statistically significant differences were found between ER and other groups (*p* < 0.05) using the Simpson and Shannon metrics.

Based on the OTU data, we examined the bacterial richness and diversity of captive (BR), semi-captive (ER), and wild (SR) groups using ACE, the Shannon index and the Simpson index (Figure 1). The number of OTUs identified in the samples depicted species richness, as estimated by ACE. A non-parametric Kruskal-Wallis test was performed in all groups. The richness indices (ACE) revealed no significant difference (*p* > 0.05) between the wild and zoo-captive groups of macaques (Table 2). However, bacterial diversity was significantly different (*p* < 0.05) among all groups, as evaluated with the Shannon and Simpson indexes. Furthermore, Shannon indexes revealed significant differences between the captive (BR) and semi-captive (ER) groups, whereas no differences between wild (SR) and semi-captive groups (ER) were found by any of the methods.

**Table 2:** Kruskal-Wallis tests of Alpha diversity in three groups of monkeys.

**K-W test (all groups)**	**ACE**	**Shannon index**	**Simpson index**
H value	2.97	14.87	14.31
*P* value	0.23	0.00059	0.00059

### Monkeys From Different Groups Have Distinct Microbiomes

**Figure 2:**
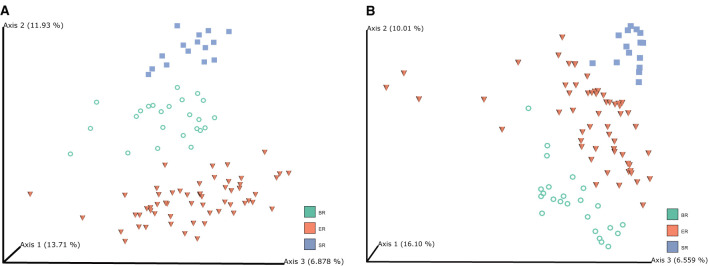
Microbiome clustering of different groups of monkeys. Principal coordinate analysis (PCoA) 3D-plots display bacterial community structure based on unweighted UniFrac distance **(A)** and Bray-Curtis distance **(B)**. The numbers of Axis 1, Axis 2 and Axis 3 showed the percent variation explained by the PCoA plots, indicating three distinctive clusters of microbiome groups.

Principal coordinate analysis (PCoA) was performed based on unweighted UniFrac (Figure 2A) and Bray-Curtis distances (Figure 2B) to visualize the dissimilarities in the bacterial communities among different groups of monkeys. The unweighted UniFrac analysis provided a much stronger clustering by population than either the weighted UniFrac or Bray Curtis distances, indicating that the clustering is likely driven by presence or absence of key taxa in different populations, rather than by shifts in the ratios of dominant members of the microbiota. In addition, PCoA plots based on Bray–Curtis distance matrices revealed that the samples from different locations formed distinct clusters, indicating that bacterial community composition conforms with the groups they were in, and hence that there were clear differences among wild, captive, and semi-captive monkeys. Analyses of distances based on relative abundance showed semi-captive groups overlapped more with captive group than with the wild group. We additionally performed Permutational Multivariate Analysis of Variance Using Distance Matrices (PERMANOVA) based on unweighted-uniFrac dissimilarity matrices (Supplementary Figure 2). Accordingly, the PERMANOVA results, indicated that (*p* = 0.001, number of permutations is 999) higher pseudo-F value in comparison of SR and ER groups with others (Supplementary Table 1).

### Comparisons of Microbial Composition Results Between 16S and WGS Approaches

**Figure 3:**
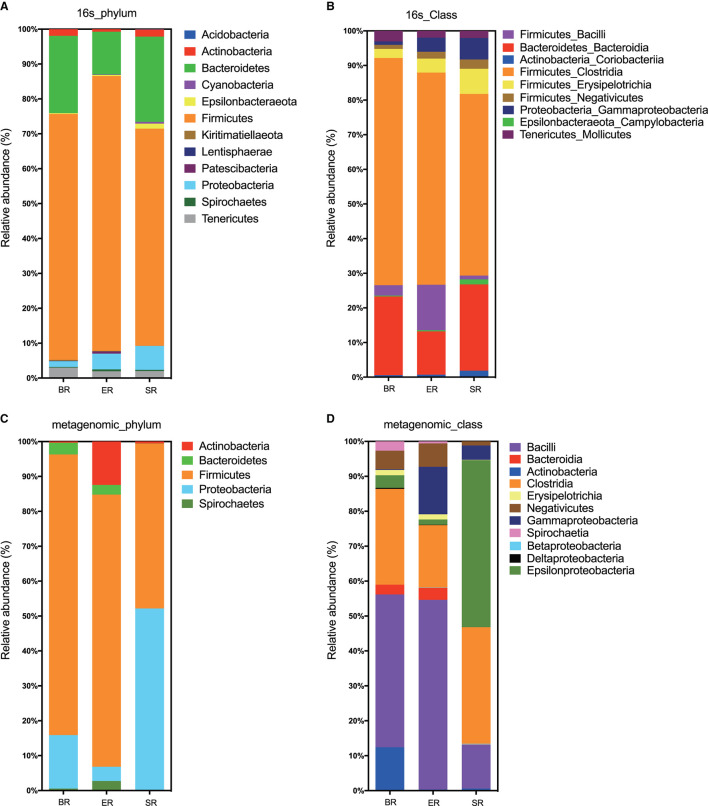
Bacterial read profiling of 16S rRNA sequencing and metagenomic approaches at phylum level **(A,C)** and class level **(B,D)**. Stacked columns for the mean of each group of samples enrolled in this study, indicating the relative abundance as a percentage of the total bacterial sequences per group. All data with an abundance of at least 0.1% in at least one subject were included.

**Figure 4:**
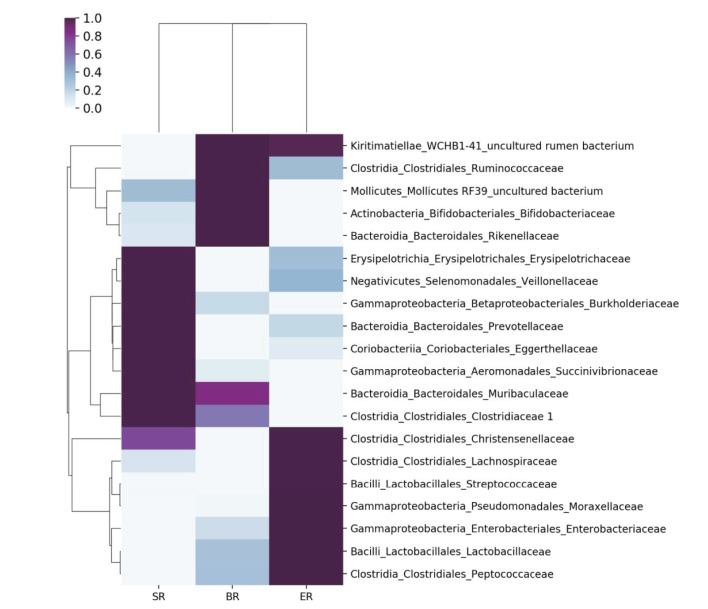
Comparisons of microbial community at the family level in different groups of monkeys. A heatmap was used to visualize the microbial composition in three groups of monkeys by 16S rRNA sequencing.

Based on 16S rRNA sequencing, the clustered operational taxonomic units identified in fecal samples were assigned to 32 bacterial phyla. Both 16S and WGS approaches identified Firmicutes, Bacteroides, Proteobacteria, and Actinobacteria as the most abundant phyla in all samples, although the proportion of Bacteroides and Proteobacteria differed substantially (Figures 3A,C). At the class level, the two approaches revealed different bacterial compositions (Figures 3B,D). The main differences lie in the Epsilonproteobacteria that only appeared at high abundance in the wild (SR) group from metagenomic sequencing, but not in the corresponding group from 16S sequencing. Furthermore, the proportion of the class Bacilli also varied greatly between the two approaches. In general, the 16S sequencing resulted in relatively consistent results across three groups, whereas WGS sequencing revealed relatively high levels of variation. Further comparisons were performed at the family level using 16S sequencing results for microbial composition between the three groups (i.e., wild, semi-captive and zoo-captive) (Figure 4).

### Detection of a Novel Simian Adenovirus in Zoo-Captive Monkeys

**Figure 5:**
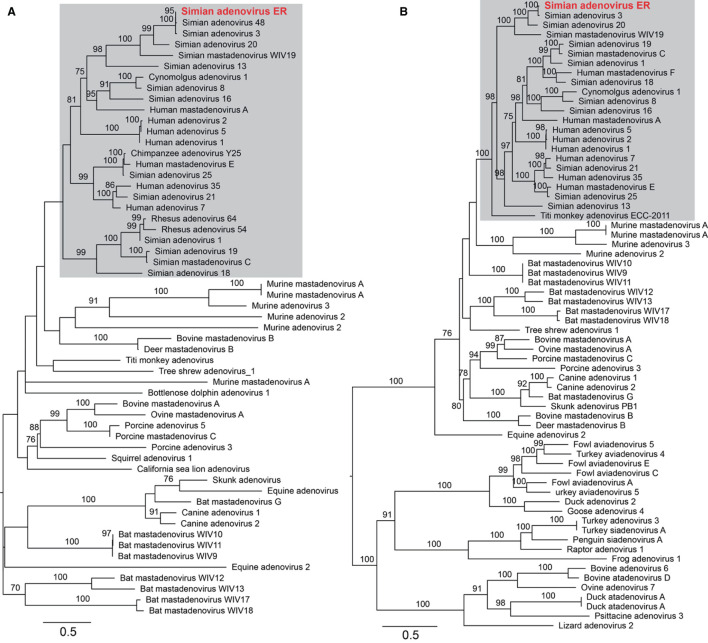
Characterization of the newly identified simian adenovirus ER in zoo-captive monkeys. Phylogenetic analysis was performed based on the amino acid sequence of E1A **(A)** and 100 K protein **(B)**. The gray shading indicates the primate adenoviruses. Branch lengths are scaled according to the number of amino acid substitutions per site. The trees were mid-point rooted for clarity only.

To assess the adenoviral reads and contigs identified from group ER, a near complete genome was derived from reassembled reads sequences that were mapped to a reference adenovirus genome (a double-strand DNA virus). The total length was 34,291 nucleotides with a GC content of 56.9%. To further characterize the adenovirus, phylogenetic trees were estimated based on a sequence alignment of the conserved region of the E1A and the 100 K protein, and utilizing reference adenovirus sequences downloaded from NCBI/GenBank. The novel virus shared 70.5% (E1A) and 88.7% (100 K) sequence identity with the closest relative—Simian adenovirus 3—within the Simian adenovirus clade (Figure 5). Based on its level of sequence divergence, the newly discovered virus likely represents a new virus species that we tentatively termed “simian adenovirus ER” (GenBank accession number: MZ062897).

### WGS-Based Characterization of the Diversity and Abundance of AMR Genes

**Figure 6:**
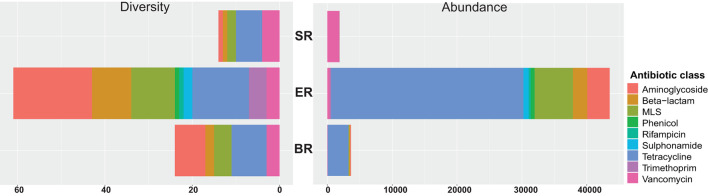
Resistome profiling of wild and zoo-captive monkeys. The diversity measures indicate the number of AMR genes detected from the ResFinder database in each class. Abundance was calculated based on the sum RPK of each class of AMR makers per metagenome.

A total of 67 acquired AMR genes were detected in the DNA-seq data sets, representing resistance against nine classes of antibiotics (Figure 6). Genes providing resistance to aminoglycosides, beta-lactams, MLS (including macrolides, lincosamides, streptogramin) and tetracyclin were found across all locations tested (Supplementary Table 2). The semi-captive group (ER) showed highest variety and abundance of antibiotic genes, followed by captive group (BR). Diversity measures indicate the number of AMR genes detected against the ResFinder database in each class. Abundance was calculated as the sum of Reads Per Kilobase of each class of AMR maker per Metagenome (RPKM) in each library. Accordingly, the wild group(SR) had the lowest variety and abundance, while genes conferring resistance to sulphonamide, rifampicin, trimethoprim and phenicol were only detected in ER group (Figure 6). In both zoo-captive groups, genes conferring resistance against Tetracycline had the highest relative abundance [i.e., AMR genes abundance (RPKM)/Total AMR genes abundance (RPKM)] at 68% in ER and 88% in BR, while genes conferring resistance against Vancomycin were the most abundant in the SR group (97.5%).

## Discussion

We present a detailed fecal microbiome analysis of the zoo-captive and wild rhesus monkeys in China. Since non-human primates are the most relevant animal models for human research, a wide range of microbial composition studies have provided important information on the features that shape host-microbiome interactions (25). To date, however, only a few studies have investigated the fecal microbiome and resistome of wild and captive primates.

Several previous studies have demonstrated that human activities such as captivity, confinement, diet and anthropogenic activity, may change the diversity and complexity of the primate gut microbiome (5). Although these studies provide evidence that captivity was associated with a reduction in diversity/richness in the gut microbiome compared to wild primates, our study revealed no such reduction, consistent with some other work (26). With respect to microbial composition, we found similarity at the phylum and class level among the three groups, but striking differences at the OTU level. The cause of such differences is still unclear. While captivity may be an important contributing factor, we are unable to exclude other factors such as geographic locations, diet and human interactions.

Notably, we used two sequencing strategies, 16S amplicon sequencing and WGS, to investigate the fecal microbiome. Generally, a similar trend of microbial composition was obtained from both approaches. However, some differences at different taxonomic levels were evident. For example, certain bacterial phyla (i.e., Tenericutes) were strongly underrepresented in shotgun WGS in comparison to 16S rRNA sequencing. Conversely, at the class level, WGS identified more diverse bacterial classes (i.e., Epsilonproteobacteria and Bacilli) than 16S sequencing, which may reflect a lack of consistent marker genes.

Due to climate change and increasing anthropogenic activities, the habitat of many wildlife species has been threatened. As such, enclosed environments like zoos provide an opportunity for intermingling of human and monkey populations (27). Previous studies have detected several zoonotic pathogens were detected in free-ranging or zoo-captive monkeys in China, such as *Escherichia coli* O98 (28), *Mycobacterium tuberculosis* (29), *Bartonella quintana* infection in captive or wild rhesus macaques (30). In addition, canine distemper virus (31), novel noroviruses, enteroviruses and enteric parasites such as *Enterocytozoon bieneusi, Cryptosporidium* spp. and *Giardia duodenalis* (32, 33) have been identified from monkeys, raising public concerns about the risk of disease transmission from zoo animals to humans. In our study, a single vertebrate-associated virus—an adenovirus—was identified in one of the zoo-captive group, ER. This virus was relatively abundant and related to the previously identified Simian adenovirus type 3. Adenoviruses have a broad host spectrum including humans and cross-species transmission have been reported in non-human primates (27, 34, 35). Furthermore, Simian adenoviruses can result in infectious respiratory and diarrheal diseases in humans, but are asymptomatic in rhesus macaques (27), indicating that they are of public health concern.

Our analysis revealed a great diversity and abundance of AMR genes in zoo-captive groups. Although AMR genes exist in nature and are transmitted among wildlife animals, habitats that are more closely linked to anthropogenic activities tend to show significantly higher levels of antimicrobial resistance (36). Common sources of AMR genes for zoo-captive groups are through contact with humans (i.e., keepers, caretakers or tourists), diet, or through receiving veterinary medication. Interestingly, the highest level of antimicrobial resistance was observed in semi-captive monkeys (ER) rather than captive animals (BR), despite the fact that the latter are more subject to human interventions. However, since the study is limited in sampling size and locations, this needs to be examined with more data in the future studies.

We identified the AMR genes *Van*G, *Van*T-G, and *Van*XY-G genes in all groups of monkeys. These confer Vancomycin resistance in gram-positive cocci such as *Enterococcus faecalis* (37). Since the first vancomycin-resistant enterococci (VRE) cases were reported in the 1980s (38), VRE-associated infections and persistent colonization in humans have raised serious public health awareness and caused huge economic impacts (39). The emergence of VREs in food-animal production systems has been largely attributed to the heavily use of avoparcin as a growth promoter (40). Even though the use of growth-promoting antibiotics in farm animals has been banned since 1997, high rates of VRE carriage have been reported globally in economic animals, as well as in companion and laboratory animals (41) as well as wildlife (42), and which might act as reservoir populations (43–54). Accordingly, the continuous long-term monitoring of a broader range of microbiome and resistomes between captive and free-ranging wildlife for enterococcal species as well as other vancomycin-resistant genes dispersal is clearly required.

In comparison to wild populations, the captive populations studied here had much higher levels of tetracycline associated resistant genes. These genes are frequently found in human isolates of the two types of bacteria that were a substantial part of the normal microbiota of primates (Firmicutes and Bacteroidetes). It was previously observed that *Enterococcus* species showed high resistance in captive black capuchin monkeys in Brazil, characterized by a higher frequency of *msr*C (95%) and *tet*(L) (57%) genes when compared to wild monkeys (55). Although we did not find *msr*C in all groups, *tet*(M) and *tet*(L) resistance genes were found at high abundance in the semi-captive group (ER); nevertheless, these AMRs genes which have also been found overlapping with existing known human gut resistomes, suggesting potential transmission via human contact with wildlife. However, because our sample size was limited future studies are needed to clarify the essential reservoirs, carriers, and vectors on the transmission chain, and to identify the factors promoting and models assessing AMR gene exchange.

## Data Availability Statement

All data generated or analyzed during this study are included in this article. All raw sequence reads were submitted to the Sequence Read Archive (SRA-NCBI) under BioProject PRJNA726842. The simian adenovirus ER was submitted to GenBank (accession number: MZ062897).

## Ethics Statement

The animal study was reviewed and approved by Institutional Animal Care and Use Committee, Sun Yat-sen University.

## Author Contributions

TJ and CZ: conceived and designed the experiments. TJ and XL: collect the samples. TJ, SZ, XL, and YY: performed the experiments. W-SC, VM, TJ, SZ, and YY: analyzed the data. W-SC, MS, TJ, and CZ: wrote the paper. SZ, W-SC, VM, and EH: revised the paper. EH, MS, and CZ: supervision. All authors contributed to the article and approved the submitted version.

## Funding

This work was funded by the Beijing Municipal Science and Technology Commission (Z141106004414051). MS was supported by Shenzhen Science and Technology Program (KQTD20200820145822023) and Guangdong Province Pearl River Talent Plan Innovation and Entrepreneurship Team Project (2019ZT08Y464).

## Conflict of Interest

The authors declare that the research was conducted in the absence of any commercial or financial relationships that could be construed as a potential conflict of interest.

## Publisher's Note

All claims expressed in this article are solely those of the authors and do not necessarily represent those of their affiliated organizations, or those of the publisher, the editors and the reviewers. Any product that may be evaluated in this article, or claim that may be made by its manufacturer, is not guaranteed or endorsed by the publisher.
