# Supplementary material for: Characterization of the Gut Microbiome and Resistomes of Wild and Zoo-Captive Macaques
Source: Front Vet Sci. 2022 Jan 24;8:778556. doi: 10.3389/fvets.2021.778556 (PMC8819141; doi:10.3389/fvets.2021.778556)
Supplement: Supplementary file 1 [file Data_Sheet_1.docx]

**Supplementary Information****Supplementary Figure 1. Rarefaction curves of different groups of monkeys**. Observed number of OTUs (A) Shannon index (B) as measures of alpha diversity. Orange: SR, wild-monkey; blue: BR, zoo-captive; cyan: ER, zoo-captive. We compared the diversity between groups using data at the full rarefaction depth (1112 sequencing depth). By dropping singleton OTUs present in each group, the SR group harboured the highest number of OTUs (172.7 ± 6.6 OTUs), indicating the greatest diversity. Followed by the ER group (164 ± 4.5 OTUs) and BR group (152.1± 4.4 OTUs). In Shannon diversity index, the SR group showed the highest value, followed by BR and ER group.


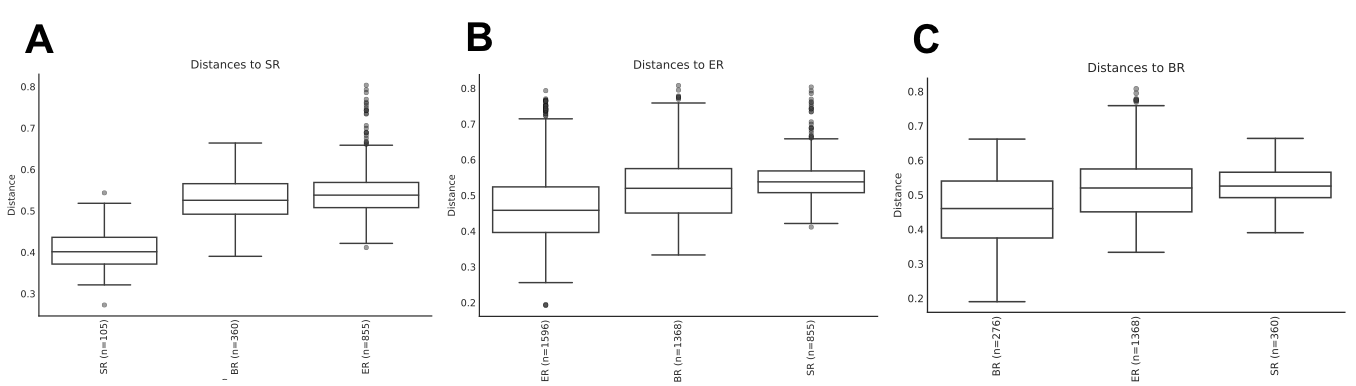


**Supplementary Figure 2.** PERMANOVA tests based on unweighted-UniFrac distances of different groups of monkeys. The PERMANOVA tests were performed based on distances to SR (A), distances to ER (B) and distances to BR (C).

**
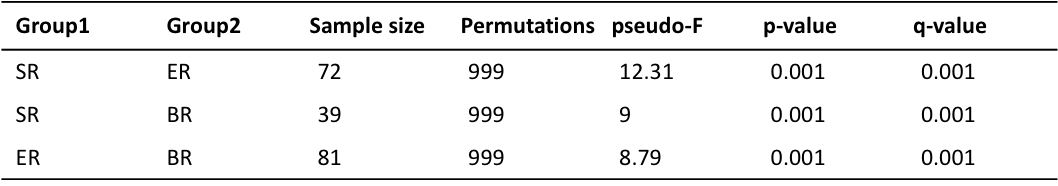
**

**Supplementary Table 1.** PERMANOVA tests based on unweighted-UniFrac distances of different groups of monkeys.

| **Group** | **Gene** | **Class** | **Abundance (RPKM)** |
| --- | --- | --- | --- |
| BR | aac(6')-aph(2'') | Aminoglycoside | 7.21 |
|  | aac(6')-Im | Aminoglycoside | 8.02 |
|  | aadE | Aminoglycoside | 50.08 |
|  | ant(6)-Ib | Aminoglycoside | 5.4 |
|  | aph(2'')-Ib | Aminoglycoside | 7.15 |
|  | aph(3')-III | Aminoglycoside | 12.64 |
|  | rmtf | Aminoglycoside | 15.16 |
|  | cfxA | Beta-lactam | 19.84 |
|  | cfxA6 | Beta-lactam | 135.32 |
|  | erm(B) | MLS | 31.37 |
|  | erm(G) | MLS | 9.25 |
|  | erm(X) | MLS | 18.3 |
|  | lnu(C) | MLS | 39.97 |
|  | tet(32) | Tetracycline | 362.87 |
|  | tet(40) | Tetracycline | 138.24 |
|  | tet(44) | Tetracycline | 137.35 |
|  | tet(O) | Tetracycline | 268.92 |
|  | tet(Q) | Tetracycline | 1240.44 |
|  | tet(W) | Tetracycline | 1056.25 |
|  | tetA(P) | Tetracycline | 11.89 |
|  | tetB(P) | Tetracycline | 11.15 |
|  | VanG | Vancomycin | 8.98 |
|  | VanT-G | Vancomycin | 37.83 |
|  | VanXY-G | Vancomycin | 7.3 |
| ER | aac(3)-Iia | Aminoglycoside | 17.39 |
|  | aac(3)-Iid | Aminoglycoside | 44.87 |
|  | aac(3)-IVa | Aminoglycoside | 16.67 |
|  | aac(6')-aph(2'') | Aminoglycoside | 241.38 |
|  | aac(6')-Ib | Aminoglycoside | 21.34 |
|  | aac(6')Ib-cr | Aminoglycoside | 23.86 |
|  | aadA1 | Aminoglycoside | 217.23 |
|  | aadA16 | Aminoglycoside | 95.46 |
|  | aadE | Aminoglycoside | 752.94 |
|  | ant(3'')-Ih-aac(6')-IId | Aminoglycoside | 108.51 |
|  | ant(6)-Ia | Aminoglycoside | 60.25 |
|  | ant(6)-Ib | Aminoglycoside | 427.88 |
|  | aph(3')-Ic | Aminoglycoside | 47.57 |
|  | aph(3')-III | Aminoglycoside | 283.66 |
|  | aph(4)-Ia | Aminoglycoside | 17.48 |
|  | ARR-3 | Rifampicin | 317.29 |
|  | blaCARB-5 | Beta-lactam | 16.96 |
|  | blaOXA-134 | Beta-lactam | 99.18 |
|  | blaOXA-212 | Beta-lactam | 18.53 |
|  | blaOXA-235 | Beta-lactam | 32.63 |
|  | blaOXA-278 | Beta-lactam | 33.4 |
|  | blaOXA-335 | Beta-lactam | 61.51 |
|  | blaOXA-360 | Beta-lactam | 50 |
|  | cfxA3 | Beta-lactam | 28.84 |
|  | cfxA6 | Beta-lactam | 1892.42 |
|  | dfrA1 | Trimethoprim | 149.51 |
|  | dfrA18 | Trimethoprim | 20.32 |
|  | dfrA27 | Trimethoprim | 39.52 |
|  | dfrG | Trimethoprim | 18.42 |
|  | erm(B) | MLS | 2421.67 |
|  | erm(C) | MLS | 62.62 |
|  | erm(F) | MLS | 229.91 |
|  | erm(G) | MLS | 661.49 |
|  | floR | Phenicol | 598.01 |
|  | lnu(A) | MLS | 26.49 |
|  | lnu(B) | MLS | 18.37 |
|  | lnu(C) | MLS | 1412.1 |
|  | mef(A) | MLS | 126.57 |
|  | mph(E) | MLS | 409.43 |
|  | msr(E) | MLS | 532.29 |
|  | rmtf | Aminoglycoside | 134.77 |
|  | strA | Aminoglycoside | 452.15 |
|  | strB | Aminoglycoside | 474.57 |
|  | sul1 | Sulphonamide | 349.1 |
|  | sul2 | Sulphonamide | 456.46 |
|  | tet(32) | Tetracycline | 1041.74 |
|  | tet(39) | Tetracycline | 1106.69 |
|  | tet(40) | Tetracycline | 4805.04 |
|  | tet(44) | Tetracycline | 2380.67 |
|  | tet(A) | Tetracycline | 21.09 |
|  | tet(L) | Tetracycline | 397.53 |
|  | tet(M) | Tetracycline | 409.39 |
|  | tet(O) | Tetracycline | 3352.56 |
|  | tet(Q) | Tetracycline | 9208.59 |
|  | tet(W) | Tetracycline | 6798.9 |
|  | tet(X) | Tetracycline | 61.66 |
|  | tet(Y) | Tetracycline | 50.25 |
|  | tetA(P) | Tetracycline | 20.27 |
|  | VanG | Vancomycin | 80.57 |
|  | VanT-G | Vancomycin | 237.52 |
|  | VanXY-G | Vancomycin | 46.45 |
| SR | rmtf | Aminoglycoside | 14.01 |
|  | cfxA6 | Beta-lactam | 1.98 |
|  | erm(B) | MLS | 2.76 |
|  | lnu(C) | MLS | 4.31 |
|  | tet(40) | Tetracycline | 5.16 |
|  | tet(44) | Tetracycline | 4.24 |
|  | tet(O) | Tetracycline | 2.73 |
|  | tet(Q) | Tetracycline | 3.41 |
|  | tet(W) | Tetracycline | 9.83 |
|  | tetB(P) | Tetracycline | 3.32 |
|  | VanG | Vancomycin | 319.34 |
|  | VanT-G | Vancomycin | 1268.34 |
|  | VanW-G | Vancomycin | 58.76 |
|  | VanXY-G | Vancomycin | 199.62 |

**Supplementary Table 2. Profiling of Antibiotic resistant genes of different groups of monkeys.**
